# Cannabidiol Attenuates Experimental Autoimmune Encephalomyelitis Model of Multiple Sclerosis Through Induction of Myeloid-Derived Suppressor Cells

**DOI:** 10.3389/fimmu.2018.01782

**Published:** 2018-08-03

**Authors:** David M. Elliott, Narendra Singh, Mitzi Nagarkatti, Prakash S. Nagarkatti

**Affiliations:** Department of Pathology, Microbiology and Immunology, University of South Carolina School of Medicine, Columbia, SC, United States

**Keywords:** experimental autoimmune encephalomyelitis, marijuana, cannabidiol, myeloid-derived suppressor cells, microRNA, multiple sclerosis, inflammation, autoimmunity

## Abstract

Multiple sclerosis (MS) is a chronic debilitating autoimmune disease without a cure. While the use of marijuana cannabinoids for MS has recently been approved in some countries, the precise mechanism of action leading to attenuate neuroinflammation is not clear. We used experimental autoimmune encephalomyelitis (EAE), a murine model of MS, to explore the anti-inflammatory properties of cannabidiol (CBD), a non-psychoactive cannabinoid. Treatment with CBD caused attenuation of EAE disease paradigms as indicated by a significant reduction in clinical scores of paralysis, decreased T cell infiltration in the central nervous system, and reduced levels of IL-17 and IFNγ. Interestingly, CBD treatment led to a profound increase in myeloid-derived suppressor cells (MDSCs) in EAE mice when compared to the vehicle-treated EAE controls. These MDSCs caused robust inhibition of MOG-induced proliferation of T cells *in vitro*. Moreover, adoptive transfer of CBD-induced MDSCs ameliorated EAE while MDSC depletion reversed the beneficial effects of CBD treatment, thereby conclusively demonstrating that MDSCs played a crucial role in CBD-mediated attenuation of EAE. Together, these studies demonstrate for the first time that CBD treatment may ameliorate EAE through induction of immunosuppressive MDSCs.

## Introduction

Multiple sclerosis (MS) is a chronic autoimmune disease in which inflammatory lesions cause damage to the myelin sheath coating the nerve fibers in the central nervous system (CNS) leading to symptoms ranging from numbness in a limb to paralysis (1). While the exact etiology of MS is unknown, studies have identified that myelin antigen-specific Th1 and Th17 cells from the periphery cross the blood–brain barrier and trigger neuroinflammation ultimately leading to destruction of myelinated neuronal cells and producing paralysis (2–9). The debilitating consequences of MS, and the current lack of effective treatment modalities, have warranted continued research into therapeutic interventions of the disease.

Marijuana cannabinoids have been shown to exhibit potent anti-inflammatory properties and have been shown to be effective in the treatment of a number of autoimmune diseases, including MS (10–12). In addition, cannabidiol (CBD), the major non-psychoactive cannabinoid component of marijuana, has also been shown to exert the neuroprotective effects (13, 14). Such studies have led to the introduction of drugs such as Sativex, which consists primarily of THC and CBD, to alleviate neuropathic pain and spasticity against MS. It is likely that ability of CBD to reduce neuropathic pain and spasticity may be independent of its anti-inflammatory effects. In addition, it is critical to identify the mechanisms through which CBD suppresses neuroinflammation in MS.

While it is well established that experimental autoimmune encephalomyelitis (EAE) pathogenesis is regulated by Tregs, recent studies have suggested that myeloid-derived suppressor cells (MDSCs) may also play a critical role in suppressing neuroinflammation (15). MDSCs are suppressor cells of myeloid lineage that were originally identified in tumor-bearing patients and models of cancer (16). In cancer, MDSCs are believed to drive T cell dysfunction resulting in promotion of tumor growth and metastasis (17–19). More recently, MDSCs have also been shown to be induced at sites of inflammation (20–22), thereby suggesting that they may play a regulatory role to temper down the inflammatory response (10, 12, 23). In autoimmune disease, MDSCs serve as attractive targets for suppressing autoreactive T cell activation and function (24). Interestingly, recent studies from our lab demonstrated that cannabinoids, including CBD, when administered into mice, induce massive numbers of MDSCs that are highly immunosuppressive (10, 25). Given that the mechanistic role of CBD-mediated neuroprotection in MS is poorly understood, we investigated whether CBD conferred a suppressed inflammatory response by triggering increased MDSCs and consequent T cell suppression.

Using EAE as a model of MS, we determined the effect of treatment with CBD on neuroinflammation, specifically focusing on the role of MDSCs. We found that CBD attenuated disease progression primarily *via* induction of MDSCs inasmuch as depletion of MDSCs could partially reverse disease mitigation, and adoptive transfer of CBD-induced MDSCs into naïve mice protected them from developing EAE.

## Materials and Methods

### Animal Use and Care

Female C57BL/6 mice were purchased from the National Institutes of Health (NIH) (Bethesda, MD, USA). All animals were housed in the University of South Carolina Animal Facility (Columbia, SC, USA). All animal procedures were performed according to the NIH guidelines under protocols approved by the Institute of Animal Care and Use Committee of the University of South Carolina.

### Reagents

The reagents used in this study were purchased as described: CBD (NIH, Bethesda, MD, USA), myelin oligodendrocyte glycoprotein (MOG35–55) peptide, H-MEVGWYRSPFSRVVHLYRNGK-OH (PolyPeptide Laboratories, San Diego, CA, USA), RBC lysis buffer, propidium iodide, hematoxylin and eosin (Sigma-Aldrich, St. Louis, MO, USA), RPMI 1640, l-glutamine, HEPES, phosphate-buffered saline (PBS), and fetal bovine serum (VWR, West Chester, PA, USA), Percoll (GE Healthcare Life Sciences, Pittsburgh, PA, USA).

### Induction of EAE and CBD Treatment Regimen

Experimental autoimmune encephalomyelitis was induced in groups of 10 female C57BL/6 mice (6–8 weeks old) as described previously (23, 26, 27). Briefly, we injected 100 µL of 150 µg MOG35–55 peptide emulsified in complete Freund’s adjuvant (Difco, Detroit, MI, USA) containing 4 mg/mL killed *Mycobacterium tuberculosis* (strain H37Ra; Difco), subcutaneously. Following immunization, 200 ng of pertussis toxin (List Labs, Campbell, CA, USA) was injected i.p. into mice on day 0, followed by a 400 ng pertussis toxin intraperitoneally (i.p.) injection on day 2. CBD (20 mg/kg; 16% DMSO:PBS) was administered daily starting at day 9 through day 25 by i.p. route. EAE mice treated with vehicle were depicted as EAE-VEH and those that received CBD as EAE-CBD.

Clinical scores (0, no clinical signs; 1, limp tail; 2, partial paralysis of hind limbs; 3, complete paralysis of hind limbs or partial hind and front limb paralysis; 4, tetraparalysis; 5, moribund; 6, death) were recorded on a daily basis. The mean score was calculated for each group every day. Each experiment was repeated at least twice with consistent results.

### Studies Using MDSCs

Myeloid-derived suppressor cells were isolated from the peritoneal cavity of mice injected with CBD, as described (28) and 4 × 10^6^ cells were injected i.p. for adoptive transfer. Splenocytes from naïve mice served as controls. To deplete MDSCs *in vivo*, we used anti-Gr-1 Abs (RB6-8C5) or isotype control Ab given 3 h after CBD injection at 0.1 mg every 48 h.

### Cytokine Detection in Serum and *Ex Vivo* Splenocytes Cultures

Experimental autoimmune encephalomyelitis mice were bled on day 16 after MOG35–55 immunization and serum was separated. Also, supernatants from cultures of splenocytes activated *in vitro* with MOG were collected after the 72 h culture. Cytokine levels for IFNγ, IL-10, IL-17, and TNFα were determined for serum and culture supernatants. All cytokines were measured using BioLegend ELISA Max kits (San Diego, CA, USA), as described in Busbee et al. (29).

### Staining Cells With Antibodies and Use of Flow Cytometry

Cells were stained with fluorescent conjugated antibodies and analyzed using the Beckman Coulter FC500 (Indianapolis, IN, USA) to determine phenotypes of infiltrating cells in the CNS. Antibodies used: fluorescein isothiocyanate (FITC)-conjugated anti-mouse CD4 (L3T4) (clone GK1.5; rat IgG2b), FITC-conjugated anti-mouse Ly-6G/Ly-6C (Gr-1) (clone RB6-8C5; Rat IgG2b), Phycoerythrin (PE)-conjugated anti-mouse/human CD11b (clone M1/70; Rat IgG2b), Allophycocyanin anti-mouse CD8 (Ly-2) (clone 53-6.7; rat IgG2a), and PE anti-mouse CD3ε (clone 145-2C11; hamster IgG).

### Cell Culture

Cell cultures were maintained in complete RPMI 1640 media supplemented with 10% heat-inactivated fetal bovine serum, 10 mM HEPES, 10 mM l-glutamine, 50 µM β-mercaptoethanol, and 100 µg/mL penicillin/streptomycin at 37°C and 5% CO_2_.

### *Ex Vivo* MOG35–55 Restimulation

Splenocytes from naïve, EAE-VEH, or EAE-CBD mice were isolated 16 days after immunization and cultured in a 96-well plate in the presence of 30 µg/mL MOG35–55 for 3 days. Supernatants were collected for cytokine analysis. Prior to harvest, splenocytes were stimulated with ionomycin, phorbol myristate acetate, Golgi-Plug for 4–6 h using Leukocyte Activation Cocktail (BD Biosciences).

### Isolation of CNS Infiltrating Cells

Experimental autoimmune encephalomyelitis-induced mice were given vehicle, or CBD as indicated earlier. On day 16, blood was collected and serum was isolated for cytokine/chemokine analysis. Spleen and inguinal lymph nodes were excised prior to perfusion. Mice were then perfused with 10 mL heparinized PBS, and whole brain and spinal cord tissue were isolated. Tissues were homogenized separately into a single-cell suspension and subjected to red blood cell lysis. Mononuclear cells from whole brain and spinal cord homogenates were isolated using 33% Percoll, as described in Rouse et al. (27). Cells were counted and stained with fluorescently tagged Abs as indicated. Absolute cell count was calculated using the following equation: Total cells bearing a specific maker = Percentage of cells with the marker as analyzed by flow cytometry × absolute number of cells/100.

### Isolation of MDSCs

Sixteen hours after CBD injection, mice were euthanized, and the peritoneal exudate was collected. In brief, the peritoneal cavity was washed three times with ice-cold 1× PBS (5 ml/wash) for 5 min with agitation to recover cells. The cells were resuspended in 1 mL, treated with Fc block for 10 min, and labeled with PE-conjugated anti-Gr1. The EasySep-positive PE selection kit (STEMCALL Technologies, Vancouver, BC, Canada) procedure was followed to isolate Gr1^+^ cells, as described previously. After isolation, cells were labeled with FITC-conjugated anti-CD11b and assessed for purity using flow-cytometric analysis (12, 30). We have indicated the use of purified Gr1^+^CD11b^+^ MDSCs in such instances in which the cells have been enriched *via* positive selection.

### Statistics

Statistical analysis was performed using GraphPad Prism 5.0 (San Diego, CA, USA). For EAE experiments, we used groups of 10 mice and figures are representative of at least two independent experiments. The clinical scores of EAE mice, at various time points, were compared between various groups using Mann–Whitney test, as described (27). In *in vitro* experiments, the data used in each figure represent mean ± SEM of at least three experiments. Statistical difference was calculated using ANOVA and Student’s *t*-test, and where stated *post hoc* analysis was performed *via* Tukey’s method. A value of <0.05 was considered statistically significant.

## Results

### CBD Attenuates EAE

**Figure 1:**
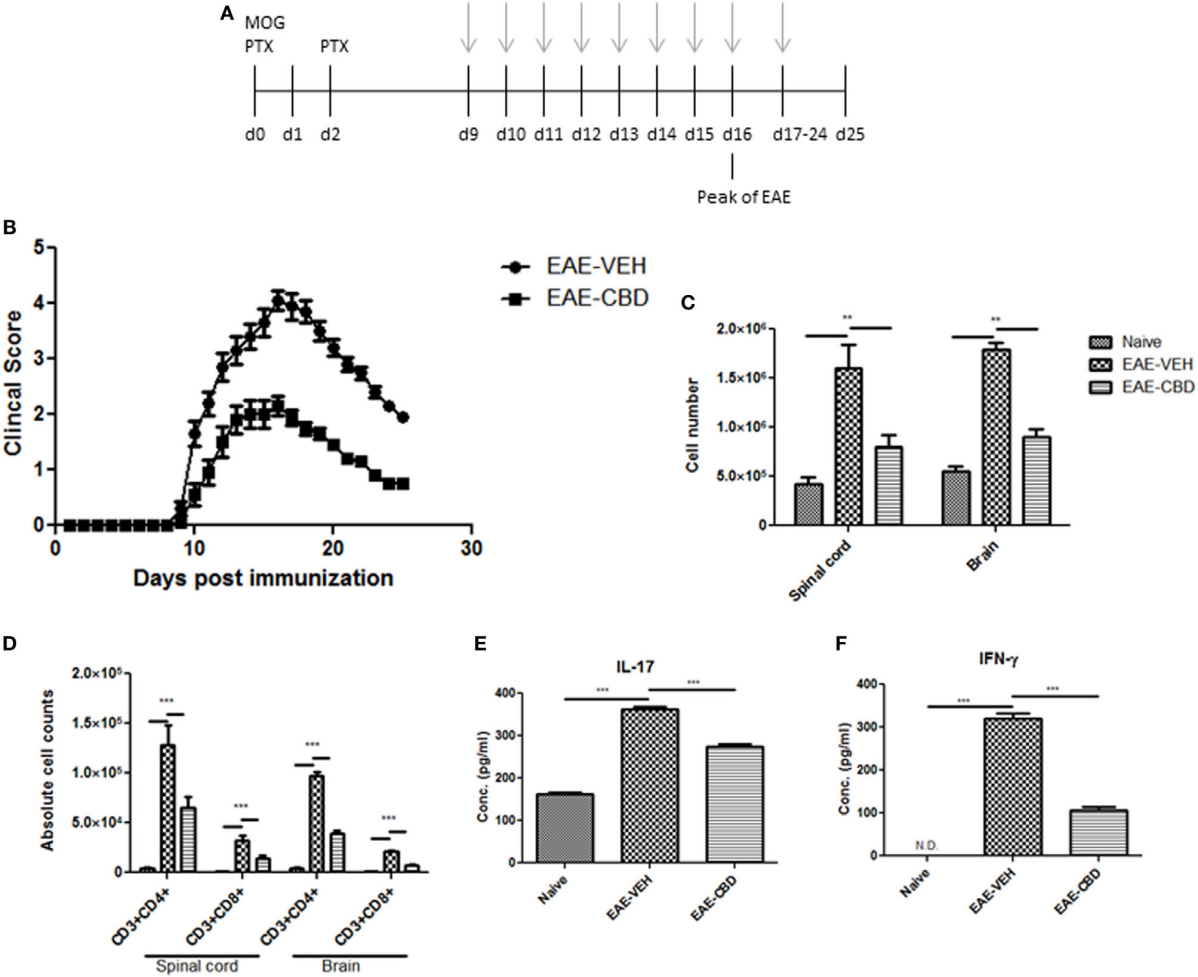
Effect of cannabidiol (CBD) treatment on the development of experimental autoimmune encephalomyelitis (EAE) in C57BL/6 mice. **(A)** Time line schematic of studies. CBD treatment was administered at time points with gray arrows. Data were assessed at peak of disease unless otherwise stated. **(B)** Clinical scores (*n* = 10 mice per group); data were presented as mean ± SEM and analyzed for significance using Mann–Whitney *U* test. Comparisons were considered significant at *p* ≤ 0.05, denoted as *. Data are representative of at least two independent experiments; in each experiment, disease incidence was 100% for each group. **(C)** Total mononuclear cell infiltrates in central nervous system. **(D)** Absolute cell counts for CD3^+^CD4^+^ T cells and CD3^+^CD8^+^ T cells; mononuclear cells stained with corresponding Abs and then enumerated using total cell count and frequency from flow cytometry. **(E)** Serum expression level of pro-inflammatory cytokines IL-17 and IFNγ analyzed by ELISA. In panels **(D–F)**, all data represented as mean ± SEM. ANOVA, ***p < 0.0001, and **p < 0.001 with Tukey’s *post hoc* test.

To study the effect of CBD on EAE, we induced EAE using MOG as an antigen and treated the mice with vehicle (EAE-VEH) or CBD (EAE-CBD) as shown in Figure 1A. CBD (20 mg/kg) treatment of EAE mice was started at the onset of clinical signs (day 9) and given every day until end of study (day 25) (Figure 1A). EAE-VEH mice progressively developed EAE disease as seen from clinical scoring (Figure 1B) with 100% incidence and maximum mean score of 4.1 ± 0.17. Treatment with CBD delayed the onset of disease and significantly attenuated clinical signs of EAE (maximum score of 2.2 ± 0.16) (Figure 1B). Moreover, the significant increase in mononuclear cell infiltrates seen in the spinal cords and brains of EAE-VEH mice was attenuated upon CBD treatment (Figure 1C). In addition, CBD treatment significantly reduced the elevated CD3^+^CD4^+^ and CD3^+^CD8^+^ cell numbers seen in the CNS of EAE-VEH mice (Figure 1D). Serum collected from EAE-VEH mice displayed elevated levels of IFNγ and IL-17 while CBD treatment significantly reduced these inflammatory cytokine levels (Figure 1E).

### Effect of CBD Treatment on Pro- and Anti-Inflammatory Cytokines and Transcription Factors

**Figure 2:**
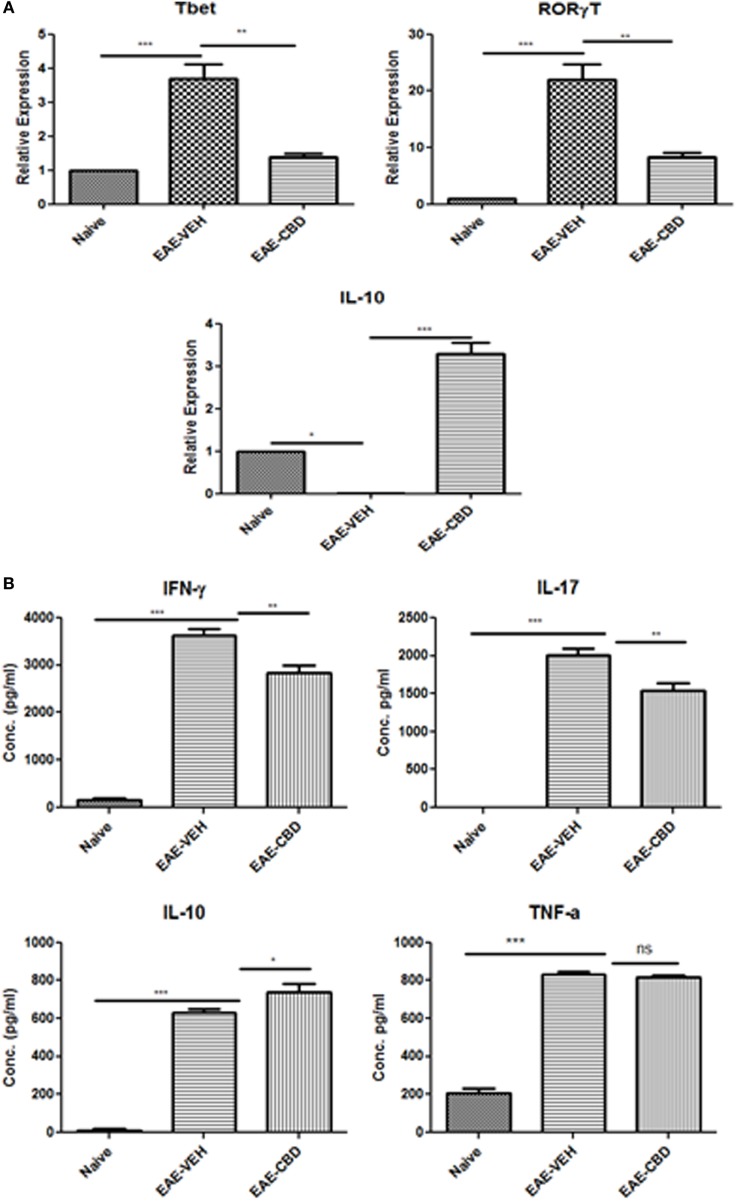
Expression profile in splenic CD4^+^ T cells from experimental autoimmune encephalomyelitis (EAE) mice. **(A)** Splenocytes were isolated from naïve, EAE-VEH, and EAE-cannabidiol (CBD) on day 16 and CD4^+^ T cells were purified using MACs selection kit. Total RNA was isolated and samples were analyzed for T-bet, IL-10, and RORγT. Data are representative of at least two independent experiments; in each experiment, *n* = 3–5 mice per group. **(B)** Splenocytes from naïve (*n* = 4), EAE-VEH (*n* = 4), and EAE-CBD (*n* = 4) were isolated 16 days after disease induction. Cells were restimulated with MOG35–55 peptide (30 μg/mL) for 3 days. Supernatants were collected and analyzed for IFNγ, IL-17, IL-10, and TNFα. Data represented as mean ± SEM. ANOVA, ***p < 0.0001, **p < 0.001, and *p < 0.05 with Tukey’s *post hoc* test.

Because EAE is triggered primarily by Th1 and Th17 cells, we next studied the effect of CBD on the cytokines related to these cells. In addition, we also studied the effect of CBD on certain critical transcription factors and cytokines: Tbx21 (T-bet), RORγT, and IL-10. To that end, splenic CD4^+^ T cells were purified from naïve, EAE-VEH, and EAE-CBD mice on day 16, and total RNA was isolated. EAE-VEH mice had significantly elevated T-bet and RORγT compared to naïve mice and treatment with CBD, significantly reduced these levels. Moreover, expression of IL-10 was significantly increased in EAE-CBD mice when compared to EAE-VEH mice (Figure 2A). To further elucidate the effect of CBD on MOG-specific T cell activation and function, we cultured splenocytes from naïve, EAE-VEH, and EAE-CBD mice, *in vitro*, in the presence of MOG35–55 peptide for 3 days. Supernatants from cells of EAE-VEH mice restimulated with MOG displayed increased IFNγ, IL-17, TNFα, and IL-10 cytokine levels, while similar cells from EAE-CBD mice produced significantly less IFNγ and IL-17, and increased IL-10 production, while producing similar levels of TNFα (Figure 2B). Together, these data demonstrated that CBD treatment promoted anti-inflammatory cytokines and transcription factors while decreasing the pro-inflammatory.

### Treatment With CBD Leads to Induction of MDSCs and Suppression of MOG-Specific T Cell Proliferation

**Figure 3:**
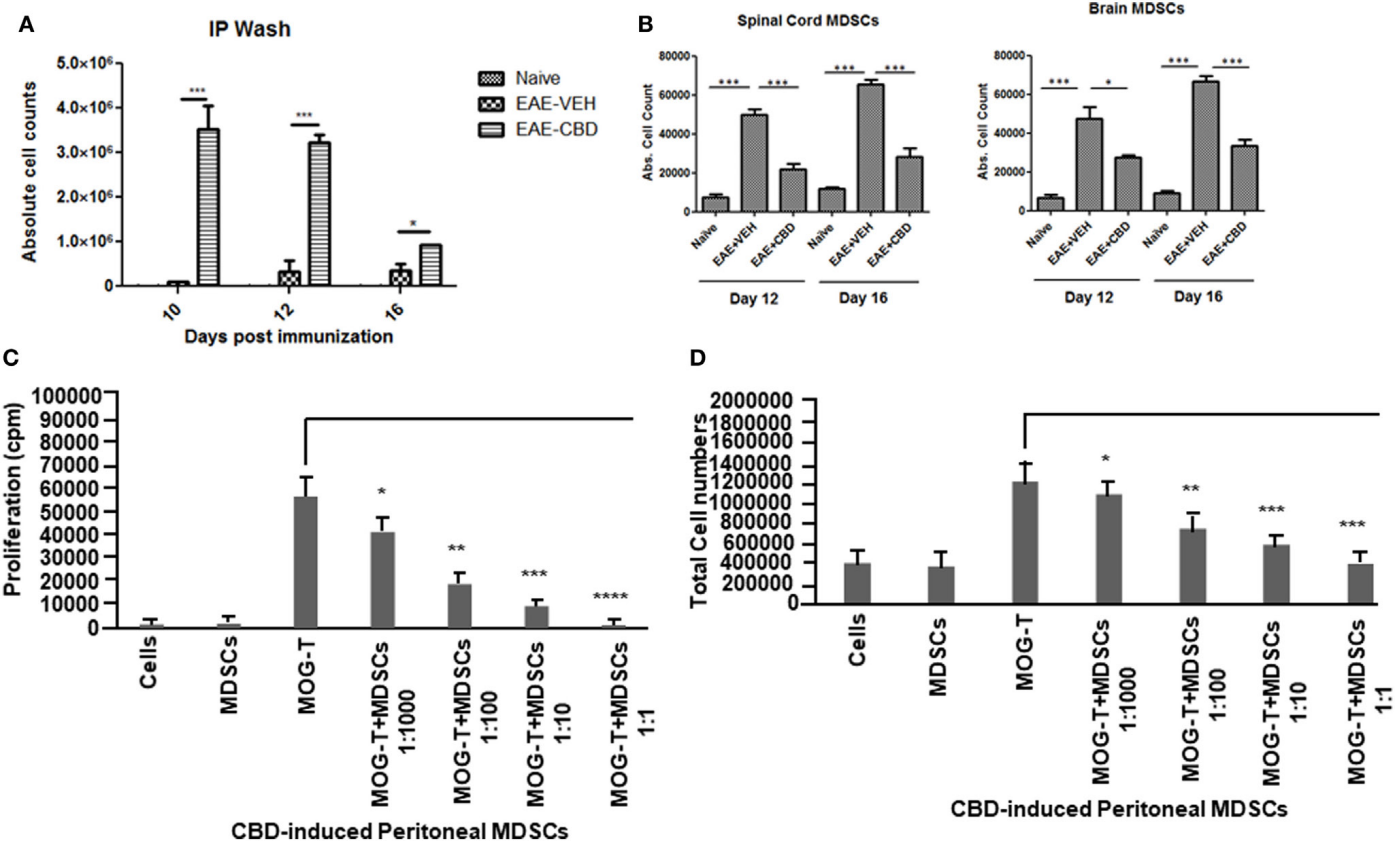
Altered expression profile of myeloid-derived suppressor cells (MDSCs) following cannabidiol (CBD) treatment. Absolute cell counts for MDSC: cells were isolated from intraperitoneal lavage **(A)**, spinal cord and brain **(B)** on indicated day. Cells were stained with CD11b and Gr-1 (MDSC) then enumerated using total cell count and frequency from flow cytometry. Data represented as mean ± SEM (*n* = 3–5 per sample). Suppressive function of CBD-induced MDSCs was tested using *ex vivo* restimulation of experimental autoimmune encephalomyelitis (EAE)-VEH splenocytes in the presence of MOG35–55 (30 μg/mL) for 3 days. Cells were pulsed with thymidine and analyzed using BetaScint Counter and were assessed for proliferation **(C)** and total viable cell number **(D)**. Data represented as mean ± SEM. ANOVA, ***p < 0.0001, **p < 0.001, and *p < 0.05 with Tukey’s *post hoc* test.

**Figure 4:**
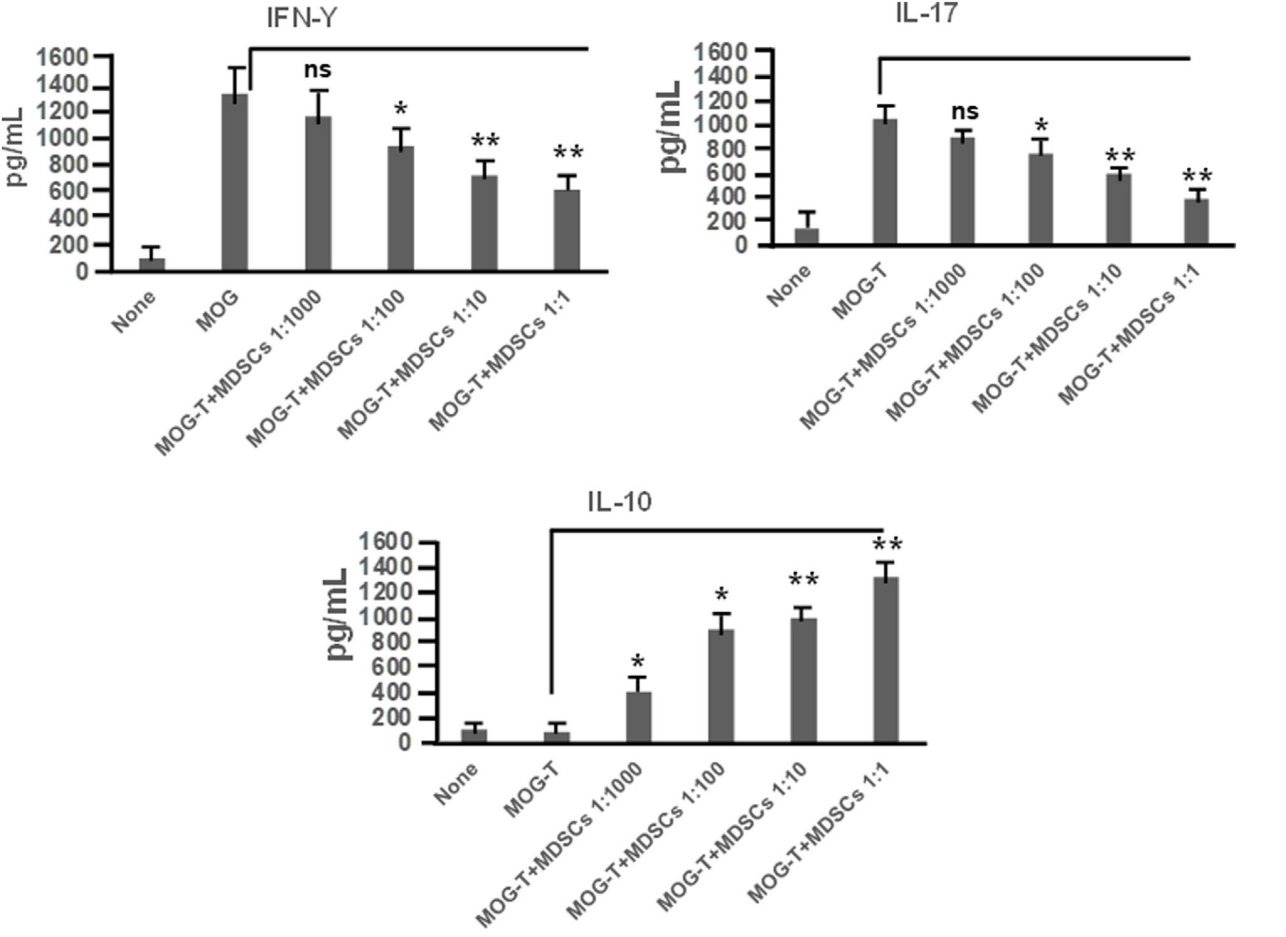
Cannabidiol (CBD)-induced myeloid-derived suppressor cells (MDSCs) alter MOG-stimulated T cell inflammatory cytokine secretion. Regulatory function of CBD-induced MDSCs was tested using *ex vivo* restimulation of experimental autoimmune encephalomyelitis (EAE)-VEH splenocytes in the presence of MOG35–55 (30 μg/mL) for 3 days, as detailed in Figure 3 legend. Culture supernatants were collected and analyzed for IFNγ, IL-17, and IL-10. Data represented as mean ± SEM. ANOVA, **p < 0.001, and *p < 0.05 with Tukey’s *post hoc* test.

CD11b^+^Gr-1^+^ MDSCs have been shown to play a crucial role in attenuating inflammation and our laboratory has previously reported that CBD treatment induces high levels of CD11b^+^Gr-1^+^ MDSCs that suppress autoimmune hepatitis (10). In the current study, we therefore investigated if the ability of CBD to suppress EAE was related to induction of MDSCs. Because CBD was administered by i.p. route, we enumerated the infiltration of MDSCs into the peritoneal cavity and found that there was a dramatic influx of CD11b^+^Gr-1^+^ MDSCs in EAE-CBD mice when compared to EAE-VEH mice that was demonstrable on days 10 and 12 and tapered off at day 16 (Figure 3A). Next, we determined if CBD treatment led to increase in the number of CD11b^+^Gr-1^+^ cells in the CNS. However, the data indicated that CBD treatment failed to increase the numbers of CD11b^+^Gr-1^+^ cells in the CNS and in fact, EAE-VEH mice had higher numbers of CD11b^+^Gr-1^+^ cells in the spinal cord and brain than EAE-CBD mice (Figure 3B). The decreased numbers of CD11b^+^Gr-1^+^ cells seen in EAE-CBD mice in the CNS may be due to the fact that there was dramatically decreased numbers of infiltrating cells in the CNS as shown before (Figure 1E). These data suggested that CBD induces CD11b^+^Gr-1^+^ cells in the periphery but not in the CNS. In addition to MDSCs, neutrophils have also been known to express CD11b^+^Gr-1^+^ phenotype. However, we have shown previously that CBD-induced CD11b^+^Gr-1^+^ cells are MDSCs and not neutrophils, because they are highly immunosuppressive while neutrophils are not (10). To further determine if CBD-induced CD11b^+^Gr-1^+^ cells were indeed MDSCs with immunosuppressive functions, we cultured splenocytes from EAE-VEH mice *in vitro* with MOG35–55 peptide for 3 days in the presence or absence of CBD-induced MDSCs isolated from intraperitoneal lavage and assessed their ability to suppress MOG-specific T cell proliferation. CBD-induced CD11b^+^Gr-1^+^ were found to be highly immunosuppressive and inhibited the proliferation in a dose-dependent manner, thereby confirming that they were MDSCs (Figure 3C). When we enumerated the number of viable T cells in such cultures, we found that the T cells were all viable while the total viable cell number decreased significantly (Figure 3D), thereby suggesting that MDSCs were not killing the T cells but inhibiting them from proliferating. To determine further the mechanism of suppression of T cell proliferation, we measured the levels of cytokines in these cultures and found that there was decreased production of IFNγ and IL-17, while induction of IL-10 was significantly increased, which was dependent on the number of MDSCs in culture (Figure 4). Because MDSCs are well known to produce IL-10, and our data showed dose-dependent response, together these studies suggested that CBD-induced MDSCs may reduce MOG-specific T cell proliferation, at least in part, by producing IL-10 and inhibiting inflammatory cytokines such as IFNγ and IL-17.

### Adoptive Transfer of CBD-Induced MDSCs Attenuate EAE Disease Progression

**Figure 5:**
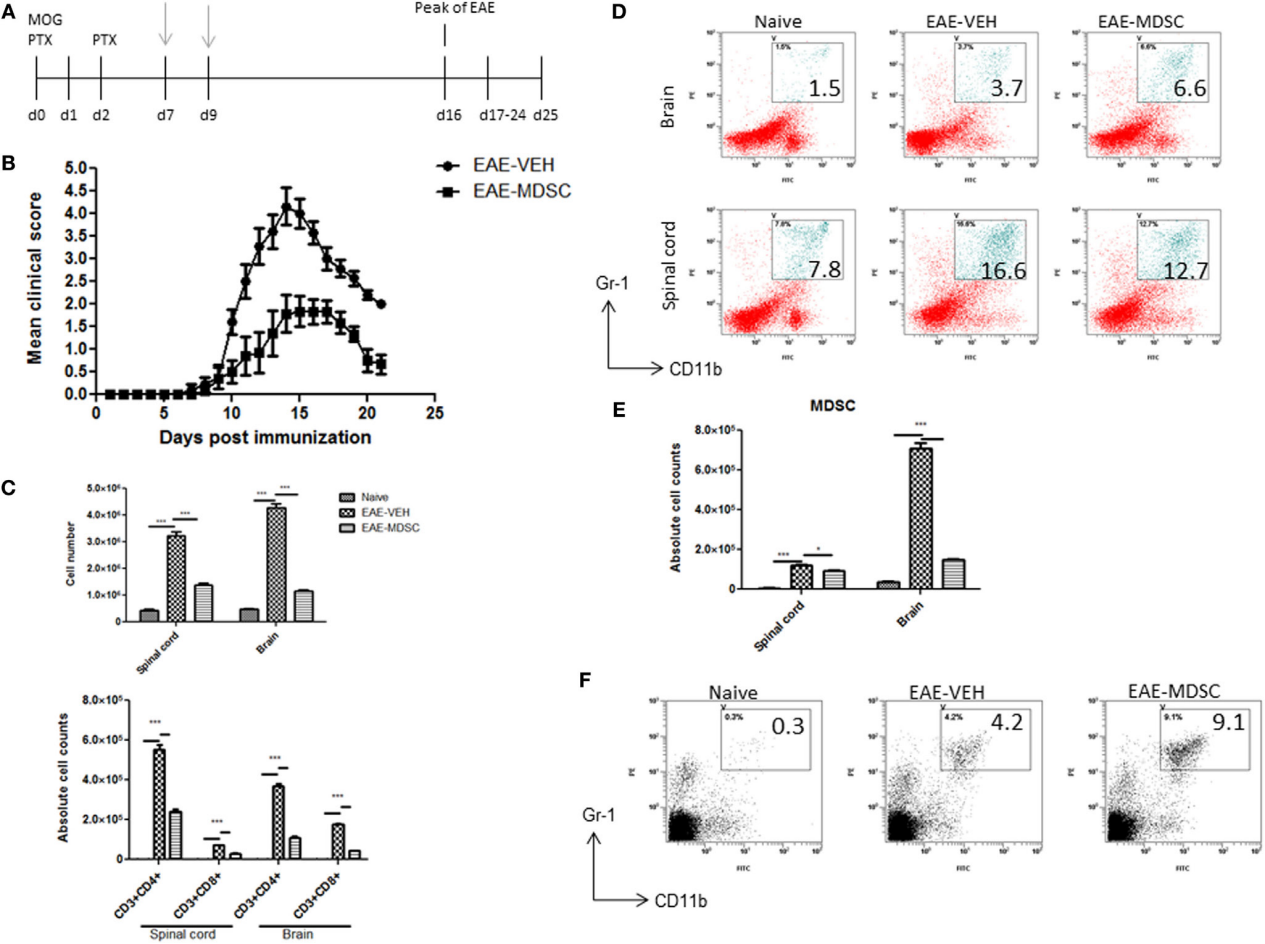
Adoptive transfer of cannabidiol (CBD)-induced myeloid-derived suppressor cells (MDSCs) attenuates experimental autoimmune encephalomyelitis (EAE). **(A)** Time line schematic of studies. CBD-induced MDSCs were administered at time points with gray arrows (d7 and d9) *via* i.p. injection. Data were assessed at peak of disease unless otherwise stated. CBD-induced MDSCs from IP cavity were collected and 4 × 10^6^ MDSCs or splenocytes from naïve C57BL/6 as a control were adoptively transferred. **(B)** Clinical scores (*n* = 5 mice per group); data were presented as mean ± SEM and analyzed for significance using Mann–Whitney *U* test. Comparisons were considered significant at *p* ≤ 0.05, denoted as *. Data are representative of at least two independent experiments; in each experiment, disease incidence was 100% for each group. **(C)** Total mononuclear cell infiltrates in central nervous system and absolute cell counts for CD3^+^CD4^+^ and CD3^+^CD8^+^ T cells; mononuclear cells stained with corresponding Abs and then enumerated using total cell count and frequency from flow cytometry. **(D)** Mononuclear cells stained with CD11b and Gr1 Abs and then analyzed by flow cytometry. Representative histograms shown. **(E)** Absolute cell counts for MDSC (CD11b^+^Gr1^+^) enumerated using total cell count and frequency from flow cytometry. **(F)** Splenocytes were stained with CD11b and Gr1 Ab and then analyzed by flow cytometry. Representative histograms shown. Vertical bars in this figure represent data collected from three to five mice per group expressed as mean ± SEM. ANOVA, ***p < 0.0001, and *p < 0.05 with Tukey’s *post hoc* test.

To confirm that CBD-induced MDSCs were in fact attenuating the clinical disease, we performed adoptive transfer experiments. To this end, we transferred purified CBD-induced MDSCs into MOG35–55 immunized mice on days 7 and 9. Four million cells were injected i.p into immunized mice, and similar numbers of splenocytes from naïve mice served as controls (Figure 5A). The transferred MDSCs were able to attenuate disease progression, as indicated by significant reduction in clinical scores (Figure 5B) and total cellular infiltration in CNS, including the numbers of CD4^+^ and CD8^+^ T cells (Figure 5C). When we assessed the MDSCs levels in the CNS tissues, we found that there was no significant increase in the percentage and absolute numbers of MDSCs in adoptively transferred mice when compared to controls (Figures 5D,E). Interestingly, however, the spleens showed a significant increase in their percentage (Figure 5F). These data together suggested that the adoptively transferred MDSCs were suppressing MOG-specific T cell activation in the periphery and not by migrating to the CNS and blocking the inflammation there.

### Attenuation of EAE by CBD Treatment Can Be Reversed With MDSC Depletion

**Figure 6:**
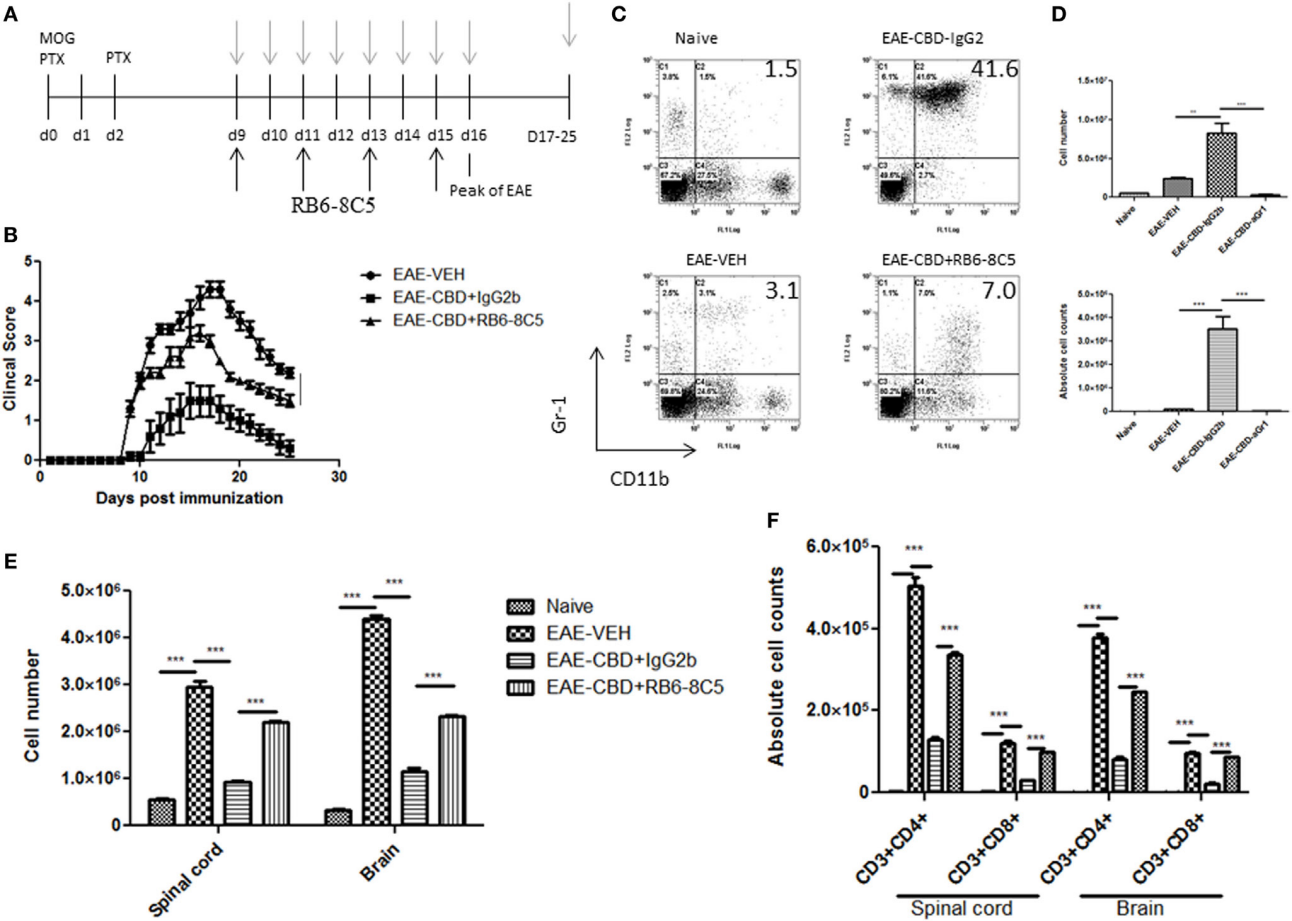
Myeloid-derived suppressor cell (MDSC) depletion negates the ameliorative effect of cannabidiol (CBD) on experimental autoimmune encephalomyelitis (EAE). **(A)** Time line schematic of studies. CBD was administered at time points with gray arrows and RB6-8C5 (anti-Gr-1) antibody was given every other day 3 h after CBD *via* i.p. injection. **(B)** Clinical scores (*n* = 5 mice per group); data were presented as mean ± SEM and analyzed for significance using Mann–Whitney *U* test. Comparisons were considered significant at *p* ≤ 0.05, denoted as *. Data were assessed at peak of disease unless otherwise stated and are representative of at least two independent experiments; in each experiment, disease incidence was 100% for each group. **(C)** On day 10, efficacy of RB6-8C5 was tested on cells recovered from peritoneal cavity. IgG2b isotype Ab was used as control. Cells were stained with CD11b and Gr1 Abs to detect MDSCs and analyzed by flow cytometry. Representative histograms shown. **(D)** Total number of cells in IP lavage and absolute cell counts for MDSC (CD11b^+^Gr1^+^) enumerated in top and bottom panels, respectively. **(E)** Total cell count of mononuclear infiltrating cells in central nervous system (CNS). **(F)** Mononuclear cells from the CNS were stained for CD3^+^CD4^+^ and CD3^+^CD8^+^ T cells for each group and absolute cell counts of these T cell subsets were enumerated. Vertical bars in this figure represent data collected from three to five mice per group expressed as mean ± SEM. ANOVA, ***p < 0.0001, and **p < 0.001 with Tukey’s *post hoc* test.

To further corroborate the role of MDSCs in CBD-mediated attenuation of EAE, we performed MDSC depletion studies using RB6-8C5 (anti Gr-1 Ab), which is highly effective in depleting such cells, as previously shown by us (12). Mice were administered CBD into EAE mice as before, and RB6-8C5 was administered every other day, 3 h after CBD treatment (Figure 6A). As an isotype control, similar concentrations of normal IgG2b Ab were used. CBD was able to significantly attenuate disease progression as seen before, and MDSC depletion reversed this effect, as indicated by clinical scores (Figure 6B). MDSC depletion was confirmed *via* peritoneal cavity lavage 18 h after CBD injection on day 9 (sacrificed day 10). MDSCs were significantly reduced in frequency and cell number following treatment with RB6-8C5 when compared to mice that were treated with control Ab (Figures 6C,D). This effect was also seen at day 16 (data not shown). Moreover, the significant reduction in total cellular infiltration and specific T cell subsets (CD4^+^ and CD8^+^) mediated by CBD was significantly reversed upon RB6-8C5 treatment (Figures 6E,F). Together, the MDSC depletion studies corroborated the adoptive transfer experiments suggesting that CBD-mediated attenuation of EAE is mediated primarily through induction of MDSCs.

## Discussion

Given that Sativex (combination of THC and CBD) is already approved for clinical use for the treatment of MS in Europe and other countries, and the recent legalization of medical marijuana in several states in the US, it is important to understand the underlying mechanism of this therapy for MS and other related inflammatory diseases. Sativex is known to reduce neuropathic pain in patients with MS. However, such effects may be independent of the anti-inflammatory properties exhibited by CBD. The anti-inflammatory benefits of CBD have been studied more recently in EAE models (31, 32). In this study, we further elucidate the effect of CBD in a model of autoimmune neuroinflammation and demonstrate for the first time that observed CBD-induced effects may be mediated by induction of MDSCs.

We chose to use a dose of 20 mg/kg body weight of CBD, which translates to 1.6 mg/kg when converted to human equivalent dose. This adds up to ~96 mg for an average human adult (60 kg). During a clinical trial, patients with Huntington disease were given 700 mg of CBD daily, for 6 weeks with no toxic effects relative to placebo (33). Also, in a recent clinical trial in epileptic children, CBD was administered at a daily dose of up to 50 mg/kg/day (34). Furthermore, based on previous studies performed by our lab, the 20 mg/kg is an optimal dose in the mouse model for suppressing inflammation and was deemed suitable and safe (10).

Consistent with other reports (32, 35), our data showed that CBD is effective at attenuating EAE, indicated by a decrease in clinical signs, delay of disease onset, diminished cellular infiltration and tissue damage in the CNS. MOG35–55 induced EAE is primarily driven by the concerted effort of Th1 and Th17 cells, producing inflammatory cytokines and effector cells to indirectly and directly damage the myelin sheath in CNS (36). In the current study, we also found systemic expression of IFNγ and IL-17 to be significantly increased at the peak of EAE. Furthermore, this effect was largely blocked with CBD treatment.

Cannabidiol has been previously shown *in vitro* to modulate Th17 responses by inhibiting IL-17 and IL-6 secretion and promoting IL-10 (11). These data are consistent with the current study inasmuch as CBD treatment *in vivo* caused significant suppression of IL-17 and IFNγ in the serum. Moreover, splenocytes from CBD-treated EAE mice that were restimulated *in vitro*, with MOG35–55, secreted less IFNγ and IL-17, while producing higher levels of IL-10. While our studies showed that MDSCs play a crucial role in inhibiting MOG-specific T cell proliferation both *in vitro* and *in vivo* using adoptive transfer, others have also noted that CBD treatment *in vitro*, may have a direct effect by inhibiting T cell proliferation or induction of apoptosis (32, 37–39). Thus, CBD may mediate its effect on inflammatory T cells both indirectly through MDSCs and directly.

Myeloid-derived suppressor cells, co-express CD11b and Gr-1 antigens and are highly immunosuppressive in nature. While other cells types have also been shown to have similar expression patterns, specifically neutrophils, we have shown previously that cannabinoids induce MDSCs rather than neutrophils (10, 12, 25, 30, 40, 41). We found that cannabinoid induced MDSCs are highly immunosuppressive while the neutrophils from the same animals were not (30). MDSCs were originally discovered in cancer patients; however, recently they have been shown to potently disrupt innate and adaptive immune responses (42). MDSCs utilize a multitude of immunosuppressive functions including; depletion of l-arginine by arginase, resulting in T cell-cycle arrest and inhibition of proliferation, expression of reactive oxygen species, secretion of IL-10, and induction of Tregs (43, 44). The role of MDSCs in EAE has at times been controversial, with some reports suggesting that circulating myeloid precursors act to perpetuate disease (45) and while others have demonstrated a regulatory role (46–48). Moreover, Ioannou et al. (47) demonstrated that patients with active MS have significantly elevated MDSCs in peripheral blood compared to healthy controls. In addition, a recent study highlighted the therapeutic potential of MDSCs in patients with MS, noting that within the MS phenotypes expression and function of MDSCs was altered (49). Because MDSCs are induced at sites of inflammation, it is not surprising that their levels are increased in the CNS during EAE, as seen in the current study as well.

In the current study, we found that following CBD injection in EAE mice, high levels of MDSCs were induced in the peritoneal cavity similar to our previous findings (10, 25). In such mice, we also noted an increase in MDSCs in the spleens but not in the CNS. In fact, in the CNS, we noted that EAE-CBD mice had lower levels of MDSCs than EAE-VEH mice. This can be explained by the fact that MDSCs induced in the periphery may not be able to migrate to the CNS. Thus, the MDSCs may inhibit MOG-specific T cell induction in the secondary lymphoid organs thereby preventing such cells from migrating into the CNS to cause the clinical disease. Therefore, we sought to elucidate this hypothesis. In doing so, we demonstrated *in vitro* that CBD-induced MDSCs were able to suppress MOG-specific T cell proliferation. Interestingly, alteration of local cytokine milieu may represent a potential mechanism for this decrease in proliferation because in such cultures with CBD-induced MDSCs, there was increased production of IL-10 and decreased induction of pro-inflammatory cytokines (IFNγ and IL-17) that was dose-dependent. IL-10 has also been shown to decrease the levels of B7 co-stimulatory molecule that is expressed on antigen-presenting cells which can bind to CD28 expressed on T cells leading to T cell activation (50–52). Thus, this could be one of the pathways through which CBD may decrease T cell activation. Second, it has been proposed that increased inflammation, particularly chronic type, induces MDSCs (43). Thus, it is also possible that in EAE-VEH mice, due to strong neuroinflammation, MDSCs are induced in higher numbers, whereas in EAE-CBD mice, due to markedly attenuated neuroinflammation, there is decreased MDSC induction. The findings that CBD induces MDSCs in the periphery but not in the CNS of EAE mice, together suggested that CBD may be acting in the periphery to attenuate MOG-specific T cell induction rather than acting directly at the CNS to decrease neuroinflammation. This observation is also supported by our findings that adoptive transfer of MDSCs failed to cause an increase of MDSCs in the CNS.

The current study conclusively demonstrated the critical role of MDSCs in CBD-mediated attenuation of EAE using both adoptive transfer experiments as well as *in vivo* depletion studies. These studies are consistent with our previous studies showing that CBD-induced MDSCs can attenuate autoimmune hepatitis (10). We also utilized RB6-8C5, an antibody against Gr-1, to deplete MDSCs while treating EAE mice with CBD. Although Gr-1 is expressed on other cells, it has been extensively used to study the effect of MDSC depletion (53–55). Our results showed that treatment with RB6-8C5 led to marked decrease in MDSCs and reversed the ability of CBD to attenuate EAE. These studies, combined with the adoptive transfer experiments conclusively demonstrated the pivotal role played by MDSCs in CBD-mediated amelioration of EAE.

In conclusion, we have demonstrated that the mitigation of EAE with CBD comes from its ability to target a range of anti-inflammatory pathways, including (i) induction of anti-inflammatory MDSCs and (ii) decrease in pro-inflammatory and induction of anti-inflammatory cytokines. Because CBD is non-psychoactive, our studies suggest that CBD may constitute an excellent candidate for the treatment of MS and other autoimmune diseases. Our studies provide further evidence of the importance of MDSCs and that manipulation of such cells may constitute novel therapeutic modality to treat MS and other autoimmune diseases.

## Ethics Statement

This study was carried out in accordance with the recommendations of Guide for the Care and Use of Laboratory Animals, National Institute of Health. The protocol was approved by the University of South Carolina Institutional Animal care and Use Committee.

## Author Contributions

Participated in research design, performed data analysis, and wrote or contributed to the writing of the manuscript: DE, MN, and PN. Conducted experiments: DE and NS. Contributed new reagents or analytic tools: MN and PN.

## Conflict of Interest Statement

The authors declare that the research was conducted in the absence of any commercial or financial relationships that could be construed as a potential conflict of interest. The reviewer, MR, and handling Editor declared their shared affiliation at the time of the review.
